# DNA record of some traditional small millet landraces in India and Nepal

**DOI:** 10.1007/s13205-016-0450-6

**Published:** 2016-06-11

**Authors:** Subramanyam Ragupathy, Shanmughanandhan Dhivya, Kirit Patel, Abiran Sritharan, Kathirvelu Sambandan, Hom Gartaula, Ramalingam Sathishkumar, Kamal Khadka, Balasubramanian C. Nirmala, A. Nirmala Kumari, Steven G. Newmaster

**Affiliations:** 1 Centre for Biodiversity Genomics, University of Guelph, Guelph, ON N1G 2W1 Canada; 2 Plant Genetic Engineering Laboratory, Department of Biotechnology, Bharathiar University, Coimbatore, Tamil Nadu India; 3 International Development Studies Program, Menno Simons College, Canadian Mennonite University, Winnipeg, MB R3C 0G2 Canada; 4 Post Graduate Department of Plant Science, Avyaiyar Government College for Women, Karaikal, 609 602 U.T. of Puducherry India; 5 Department of Anthropology, University of Manitoba, Winnipeg, R3T 2N2 Canada; 6 Local Initiatives for Biodiversity, Research and Development (LI-BIRD), P.O. Box 324, Pokhara, Kaski Nepal; 7 Department of Plant Biology and Plant Biotechnology, S.D.N.B. Vaishnav College for Women, Chromepet, Chennai, Tamil Nadu 600 044 India; 8 Centre for Biocultural Diversity, 45A Srinivasan Street, Madipakkam, Chennai, 600091 India; 9 Centre for Excellence in Millets, Tamil Nadu Agricultural University, Atthiyandal, Thiruvannamalai District, Thiruvannamalai, Tamil Nadu 606 603 India

**Keywords:** Traditional knowledge, Small millets, DNA barcoding, Tiered approach, PPVFR

## Abstract

Despite the extensive use of small millet landraces as an important source of nutrition for people living in semi-arid regions, they are presently marginalized and their diversity and distribution are threatened at a global scale. Local farmers have developed ancient breeding programs entrenched in traditional knowledge (TK) that has sustained rural cultures for thousands of years. The convention on biological diversity seeks fair and equitable sharing of genetic resources arising from local knowledge and requires signatory nations to provide appropriate policy and legal framework to farmers’ rights over plant genetic resources and associated TK. DNA barcoding employed in this study is proposed as a model for conservation of genetic diversity and an essential step towards documenting and protecting farmers’ rights and TK. Our study focuses on 32 landraces of small millets that are still used by indigenous farmers located in the rain fed areas of rural India and Nepal. Traditional knowledge of traits and utility was gathered using participatory methods and semi-structured interviews with key informants. DNA was extracted and sequenced (*rbcL*, *trnH*-*psbA* and *ITS2*) from 160 samples. Both multivariate analysis of traits and phylogenetic analyses were used to assess diversity among small millet landraces. Our research revealed considerable variation in traits and DNA sequences among the 32 small millet landraces. We utilized a tiered approach using *ITS2* DNA barcode to make 100 % accurate landrace (32 landraces) and species (six species) assignments for all 160 blind samples in our study. We have also recorded precious TK of nutritional value, ecological and agricultural traits used by local farmers for each of these traditional landraces. This research demonstrates the potential of DNA barcoding as a reliable identification tool and for use in evaluating and conserving genetic diversity of small millets. We suggest ways in which DNA barcodes could be used in the Protection of Plant Varieties and Farmers’ Rights in India and Nepal.

**Electronic supplementary material:**

The online version of this article (doi:10.1007/s13205-016-0450-6) contains supplementary material, which is available to authorized users.

## Introduction

Cultivated for centuries, millets are an important source of human food in semi-arid regions of Asia and Africa. In present day India, pearl millet and sorghum are the two major millets that undergo large-scale cultivation with commercial implications at the global level (Rai et al. 1999; Gruère et al. 2009). Unfortunately, traditional landraces of small millets have been marginalized and their distribution is threatened. Small millets are equally important as major millets, but there is a lesser known group of six species that compromise thousands of traditional landraces (Nagarajan and Smale 2007; Gruère et al. 2009). The six different species of small millets cultivated by farmers in India are as follows: little millet (*Panicum sumatrense* Roth ex Roem. & Schult.), proso millet (*Panicum miliaceum* L.), Italian millet (*Setaria italica* (L.) P. Beauv.), kodo millet (*Paspalum scrobiculatum* L.), Indian barnyard millet (*Echinochloa frumentacea* Link.), and finger millet (*Eleusine coracana* (L.) Gaertn.) (Newmaster et al. 2013a, b, c). Compared to pearl millet and sorghum, these millets have shorter slender culms and smaller grain size (Maloles et al. 2011). In Nepal, finger millet (*Elusine coracana*) is one of the commonly grown millets and an important staple crop in the hill and mountain farming systems, especially in the rainfed and marginal agricultural lands. The total area under finger millet cultivation was 268 thousand hectares with a national average productivity of 1.11 mt/ha in 2009/2010. It occupies nine percent of total cultivable land and around 75 % of finger millet cultivation areas located in the mid hills (Upreti 2002). Foxtail millet (*Setaria italica*), barnyard millet (*Echinochloa frumentacea* Link.), little millet (*Panicum miliare*) and proso millet (*Panicum miliaceum*) are other small millets grown in Nepal. Apart from the morphological differences, it is the incredible underutilization of small millets that distinguishes them from the major millets. Although these landraces have considerable utility for farmers, these “small” millets have received relatively low market and research support for enhancing crop area, production, improvement and utilization (Nagarajan and Smale 2007).

Despite having very little commercial development, small millets are grown extensively by indigenous farmers in rural southern India for their nutritious content and resistance to drought (Maloles et al. 2011). Farmers have selected for specific ecological/agricultural traits in small millet landraces that have allowed them to cultivate these crops in marginal regions (Weber 1998). This ancient breeding program is entrenched in traditional knowledge (TK) that has sustained cultures such as the *Malayali* farming communities in the Eastern Ghats (of India) for thousands of years (Rengalakshmi 2005). This traditional knowledge classifies traits into three broad categories: (1) morphological traits (plant height, seed shape, size, etc.), (2) agricultural traits (grain yield, drought tolerance, etc.), and (3) traits that have cultural value (gastronomic and medicinal traits) to the farmers (Newmaster et al. 2013a, b, c). Using quantitative morphometric analysis, Maloles et al. (2011) show that the *Malayali* TK classification is hierarchical and recognizes considerable fine scale variation in millet landraces with high consensus among farmers (Rengalakshmi 2005; Maloles et al. 2011). The TK classification is multi-tiered with a primary tier that represents five species and a secondary tier that classifies the 19 different landraces (Maloles et al. 2011). By selecting for traits that are beneficial for livelihood, farmers inadvertently imposed considerable selection pressures on the genome of small millet landraces. Traits notified millets were retained by farmers for long time—several ages, especially for their own needs and cultural value.

Both India and Nepal are parties to the Convention on Biological Diversity (CBD), which has three main objectives: the conservation of biological diversity, the sustainable use of its components and fair and equitable sharing of benefits arising out of the utilization of genetic resources. CBD envisaged that the benefits accruing from commercial use of traditional knowledge have to be shared with the people responsible for creating, refining and using this knowledge. The Article 8 (j) of the CBD provides for respecting, protecting and rewarding the Knowledge, Innovations and Practices (KIP) of local communities (Patel 2004). Although the development of an appropriate form of protection for the knowledge of local farming communities is of great interest to the developing countries including India and Nepal, there have been minimal steps in systematic documentation and analysis of indigenous knowledge of farmers. Nepal has recently started streamlining the conservation of biodiversity by putting up the Agriculture Genetic Resource Centre (Genebank) (http://narc.gov.np/org/gene_bank.php) under the government owned Nepal Agricultural Research Council (NARC). The conservation strategies followed are the ex situ conservation, on-farm conservation and in situ conservation. However, a systematic recording and documentation of DNA database is still a long journey. The PPVFR Act in India has set the tone for farmers’ ownership over plant genetic resources (Swaminathan 2002). The implementation of the PPVFR Act has led to creation of the authority that registers local landraces of different cultivated species conserved by the farmers and communities, which will facilitate their claims for benefit sharing and recognition. The next important step is to document genetic diversity using a common method. India seems to be a step ahead compared to Nepal, yet does not have a National DNA database for Indian plant species or landraces. The researchers rely on the allele frequency calculations on the western species/landraces through blast analysis of the NCBI GenBank database. The lack of any genetic database precludes the protection of plant varieties and farmers’ rights in these countries. The paper illustrates that how DNA barcoding can be used as a tool for classifying genetics and commodity identification tool can ensure authentication and traceability of landraces. This could provide a DNA-based model for conservation of genetic diversity and the associated biocultural diversity of millet landraces still used and shared among rural communities.

DNA barcoding has been shown to be an effective tool for plant identification. Plant barcoding utilizes a multiregional approach using multiple DNA regions to identify plants (Chase et al. 2005; Newmaster et al. 2006; Kress and Erickson 2007; Fazekas et al. 2008; Hollingsworth et al. 2011). The tiered approach is an efficient two-region approach that overcomes the issue of alignment with noncoding regions such as the *trnH*-*psbA* spacer or highly variable nuclear regions such as ITS2 (Newmaster et al. 2006). This approach uses *rbcL*, as the first tier because it is easily amplified and aligned, which provides a scaffold for a highly variable second tier region such as ITS2 (Newmaster et al. 2013a). Earlier recommendations for including *matK* as a core plant barcode (CBOL Plant Working Group 2009) have now been invalidated because of associated problems encountered with PCR amplification and considerable costs associated with retrieval of a *matK* DNA sequence (Von Cräutlein et al. 2011; Kuzmina et al. 2012). Current research suggests the use of *ITS2* as the second tier as proposed by Chen et al. (2010) because it provides higher species resolution (Yao et al. 2010; China Plant BOL Group 2011). Many applications have been using this *rbcL* + *ITS2* tiered approach to monitor species diversity (Von Cräutlein et al. 2011), reconstruct animal diet from scat samples (McMullin et al. 2011), discover new species (Liu et al. 2013), identify plants in ethnopharmacology (Liu et al. 2012), floristic analysis (Kuzmina et al. 2012) and to detect fraudulent market place substitution in the natural health products industry (Newmaster et al. 2013a, b, c; Nithaniyal et al. 2014).

Past research examining genetic variation in small millets has focused mostly at the subfamily level with limited research resolving variation at either the specific or intraspecific level (Salimath et al. 1995; Panwar et al. 2010; Gupta et al. 2011a, b). Recently, Maloles et al. (2011) used DNA barcoding to examine genetic variation in plastid regions *rbcL*, *trnH*-*psbA* and *matK* among 19 TK landraces, but found that these regions were invariant. Expanding on this study, Newmaster et al. (2013a, b, c) used the nuclear regions *ITS* (*ITS*, *ITS1* and *ITS2*) to examine variation between 15 landraces from rural southern India. They observed high interspecific and intraspecific variation in the ITS regions among six species of small millets. In addition, they identified 100 % accurate species and landraces from 150 blind samples and showed that genomic variation is aligned with the TK hierarchical classification. Furthermore, they also found that the plastid region *trnH*-*psbA* allowed differentiation for eight out of 15 landraces. Though classification by farmers is finer than that of scientific classification, it is impractical to employ TK as a species identification tool due to its complex and time-consuming nature. Newmaster et al. (2013a, b, c) demonstrated the potential of DNA barcoding as a reliable millet identification tool and the use of *ITS* and *trnH*-*psbA* regions as standardized genomic regions for evaluating genetic diversity among species and landraces (Newmaster et al. 2013a, b, c).

There are key uncertainties concerning the genetic and trait diversity of small millets. We have a limited understanding of TK of landraces and a very poor understanding of how much variation there is in genetic, trait and TK among the many landraces still used today. Such information can help inform breeding and sustainability programs targeted towards conserving these underutilized yet valuable crops that support many rural communities of southern Asia. The present study aims to answer the following questions to provide more information on the diversity among small millet landraces: (1) Is there a variation in traits among the small millet landraces? If so, how does this variation group landraces in multivariate ordination space?; (2) Is there a sufficient genetic variation (*ITS2*, *trnH*-*psbA*) to allow a fine scale classification of 32 small millets?; and (3) How can DNA barcodes can help in protecting farmers’ rights recognized under the CBD?

## Materials and methods

### Study sites

The sites selected for this research project were located in rain fed areas of India and Nepal, which have high incidence of poverty, food and nutritional insecurity. They are remote, underdeveloped and have a considerable indigenous population. These are areas where traditional millet landrace-based cropping systems still exist. The local farmers in these areas have considerable traditional knowledge of small millet landraces used in traditional agriculture. We conducted research in the three districts of Tamil Nadu, India and two districts in Nepal. From India, we selected (1) Plains of Peraiyur located at 9.72°N 77.8°E (Madurai district) that fall in moderately food secure category, poorer and more food insecure than their respective state’s performance; (2) Eastern Ghats of Anchetty located at 12°31′11″N77°46′48″ (Krishnagiri district), which is a predominately subsistence-based food security among small and marginal rainfed farmers due to the drought prone nature of the area, high levels of poverty, remoteness and the need to conserve the millet-based subsistence cropping system in the area 3) Jamnamarudur located at 12°35′52″N 78°53′11″E (Vellore district), which is predominately subsistence-based food security, small millets are regularly grown and consumed. In Nepal, field research was conducted in Kaski—28°16′0″N 83°53′0″E and Dhading—27°52′0″N 84°55′0″E districts from the central hills of Nepal. Farmers grew only finger millet (also known as *kodo* in local language) as ruling crop. Their preference over finger millet cultivation is mainly because of the hill top landscape, climatic adversity, and the cultural importance of finger millet-based products.

### Traditional knowledge of small millet landraces

Fieldwork was conducted between April 2011 and July 2012 with 96 research participants (called informants) consisting of small and marginal farmers. We interviewed 48 male and 48 female informants categorized into three age groups: age group 1—young people between 5 and 25 years (*n* = 20); age group 2—middle age people between 26 and 50 years (*n* = 26); age group 3: elders between 51 and 75 years (*n* = 20). Initially, a 4th age group (>76 years) was considered for knowledge stratification, but this was excluded because the sample size of participants was too small and had no consistency to be included in the study. The knowledge of these (4th age group) elders was considerable and notes were made of this elderly knowledge where appropriate within the discussion.

This study comprises the collection of two distinct types of information: millet-specific information to elicit morphological attributes of small millets for DNA barcoding and the grower/farmer-specific information to elicit sociocultural characteristics of the indigenous millet growers, traditional knowledge and importance of millets in local livelihoods and food security. This distinction is overlapping and seems to be artificial, but it serves a purpose to inform the interdisciplinary scholarship. Millet-specific information was collected using the following methods: (1) Knowledge holders were requested to accompany us to the field and identify the plants used; (2) specimens were brought to the village and shown to knowledge holders for sharing information; and (3) photographs taken from the field crops in the vicinity were also used as stimulus. These exercises were conducted using suitable participatory rural appraisal techniques such as oral history, transect walk and resource mapping. Farmer-specific data were collected using focus group discussions (FGD), individual in-depth interviews, and passive observation to document farmers’ traits knowledge on small millet cultivation, consumption and nutrition. Data were collected at different intervals of time with diverse groups of farmers. Village meetings were organized and the objectives and outcome of the study were explained in detail. Appropriate suggestions/modifications given by the villagers were incorporated. The prior informed consent was obtained from the village councils where the study was intended, as a part of ethical commitment. Also the individual respondent’s prior informed consent was obtained. Both qualitative and quantitative data collection and analysis tools were used. Research instruments included were the checklists, semi-structured interviews, photo identification of selected plants, and informal key informant interviews (Pelto and Pelto 1990; Etkin 1993; Vogl et al. 2004; Stepp and Thomas 2005). Effort was taken to attend all festivals and ceremonies happening in the village, which helped develop a better understanding of their culture, helped understand the role played by millets in people’s livelihood and also helped build rapport in the local community. Oral permission and written consent were obtained from the subjects before taking photographs. All the data collection instruments were pretested with informants that were not later represented in the sample to determine whether the questions generated the desired information (Bernard 1994). This was verified as consumed by the three socio-cultural groups—through photographs, providing a ‘correct’ vernacular name for the plants that informants could identify (secondary materials were used for identification of species names). Ethics approval was attained from the University of Guelph Research Ethics Board (REB11AU002).

### Small millet samples

Voucher specimens were collected and tagged except from millet farmers’ landscape and deposited at the Center for Biocultural Diversity Herbarium (http://www.cbdindia.org) and seed samples were deposited in “All India Coordinated Small Millet Integrated Project” (AICSMIP), Bangalore, India. Information collected was recorded in field notebooks and electronic recorder. The specimens were identified once in three months when they were brought back to the CBD head office herbarium at Chennai, with the help of the Floras such as Flora of British India (Hooker 1894), Flora of the Madras Presidency (Gamble 1915; Nair and Henry 1983; Henry et al. 1987, 1989).

### DNA extraction and PCR amplification

Total genomic DNA was isolated from 50 mg of the fresh plant material using the Nucleospin Plant II Mini DNA Extraction kit (Macherey–Nagel, Germany). The same protocol as per the manufacturer’s instructions was followed. Duplicate samples were maintained to ensure the consistency of the experiment. The isolated DNA samples were quantified by NanoDrop N-1000 spectrophotometer (NanoDrop Technologies Inc., USA). The PCR was performed under standard conditions for the primer pairs: *psbA*-*trnH*, and *ITS2* as described in Newmaster et al. (2013a, b, c).

### Sequence and phylogenetic analysis

Sequencing was carried out in two directions of each fragment with Big Dye terminator v3.1 Cycle sequencing kit (Applied Biosystems, USA) and an automated ABI 3730 sequencer (Applied Biosystems, USA). The sequences were edited and aligned using Clustal W (Thompson et al. 1994) application in Bioedit (Hall 1999). The aligned sequences were used to construct an optimal tree using MEGA 5.0 (Tamura et al. 2011) tool. The evolutionary tree was constructed using the Kimura-2-parameter model (Kimura 1980) and the Neighbor Joining tree (Saitou and Nei 1987) was constructed by resampling the tree using 1000 bootstrap replicates (Felsenstein 1985).

### Generating DNA barcodes for millet landraces

We created the project titled “Food Security Small Millet (FSSM)” in Barcode of Life Database System (BOLD systems, which is a publicly available data source). This provides a searchable public database of barcode-sequence clusters that closely approximate landraces of small millets in this study. We submitted all 32-millet landrace’s DNA sequence and millet landraces voucher information to the BOLD system, which generated the barcodes (Table 1) for all the landraces in this study and those closely related species in the BOLD database. BOLD automation includes all the other information associated with the quality of sequence, voucher information, sample collection sites including the geographic co-ordinates, which allowed BOLD to generate distribution maps for all samples.

**Table 1:**
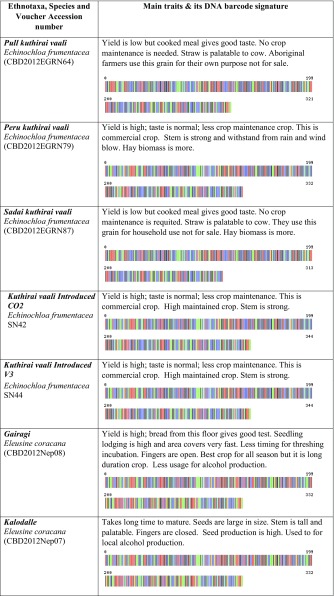

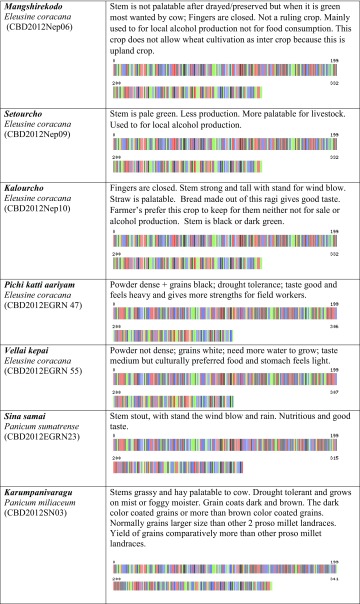

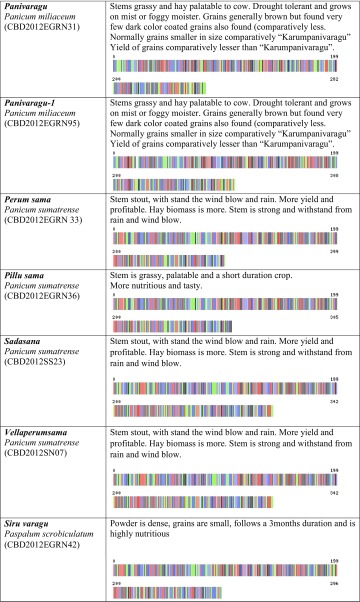

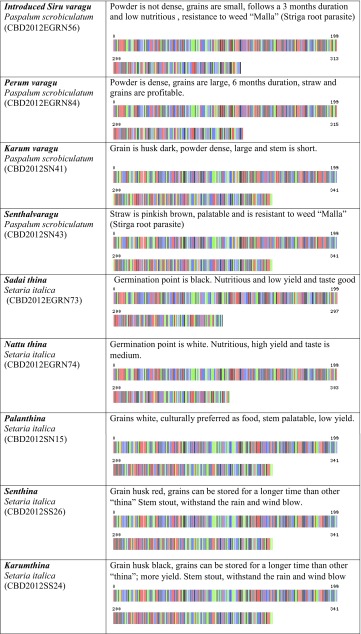

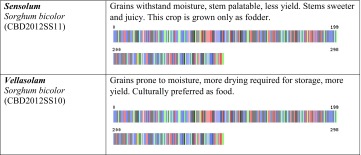
Pictorial DNA barcodes are serving a commodity identification tool to ensure authentication and traceability of millet landraces main traits of 32 small millets

Voucher accession numbers are provided for deposits at the Centre for Biocultural Diversity, Chennai, India

### Millet landrace DNA barcode ID tool

We collected 160 samples of millet seeds from farmers, representing the 32 landraces (five samples per landrace). These samples were blind labeled and randomly drawn from a pool of 160 seed packets labeled only with a number (unknown landraces). These samples were all barcoded using *ITS2* as a possible marker for quick and efficient molecular identification. Identification of unknown sample *ITS2* sequences was conducted using BLAST against a local DNA barcode library for the selected landraces with a minimum BLAST cutoff of 97 % identity for a top match. These results were verified by neighbor-joining analysis and by evaluating the branches leading to specimens tested as compared with sequences of reference species. We also used the “best match” and “best close match” functions of the program TAXONDNA (Meier et al. 2006) as an independent analysis for successful identification. We recorded cost and time to provide an estimate for use as a millet landrace DNA barcode identification tool.

### Multivariate analyses

We used multivariate analyses to explore variation among 32 landraces for 50 agricultural traits. The classification structure in the landraces to the agricultural trait characters was analyzed with nonmetric multidimensional scaling (Kruskal 1964) using “R” software (R Core Team 2012). In NMDS, the Bray–Curtis distance measure was used because of its robustness for both large and small scales on the axes. Data were standardized by species maxima and two-dimensional solutions were appropriately chosen based on plotting a measure of fit (‘stress’) to the number of dimensions. Stress represents distortion in the data and a stress value over 0.15 is high enough that the results are invalidated. One thousand iterations were used for each NMS run, using random start coordinates. The first three ordination axes were rotated to enhance interpretability with the different axes.

## Results and discussion

### Genomic variation among miner millet landraces

There is limited research on the genomic variation found among small millet landraces. Molecular techniques such as restriction fragment length polymorphism (RFLP), randomly amplified polymorphic DNA (RAPD), inter simple sequence repeat amplification (ISSR) and microsatellites have been employed to understand genetic diversity in major and various small millet species (Salimath et al. 1995; Chowdari et al. 1998; Parani et al. 2001; Gupta et al. 2010; Panwar et al. 2010). However, we have a limited understanding regarding the genetic diversity among small millet landraces that sustain farming communities in South Asian countries like India and Nepal. Unfortunately, molecular techniques used in the past (RFLP, RAPD, ISSR) are difficult to reproduce and are not cost-effective tools for identification and conservation of genetic diversity of small millet landraces. Earlier research published by Maloles et al. (2011) and Newmaster et al. (2013a, b, c) evaluated variation among 19 landraces using plastid regions and 15 landraces using nuclear landraces, respectively. Our current research expands on these studies to evaluate variation among 32 landraces in India and Nepal.

**Fig. 1:**
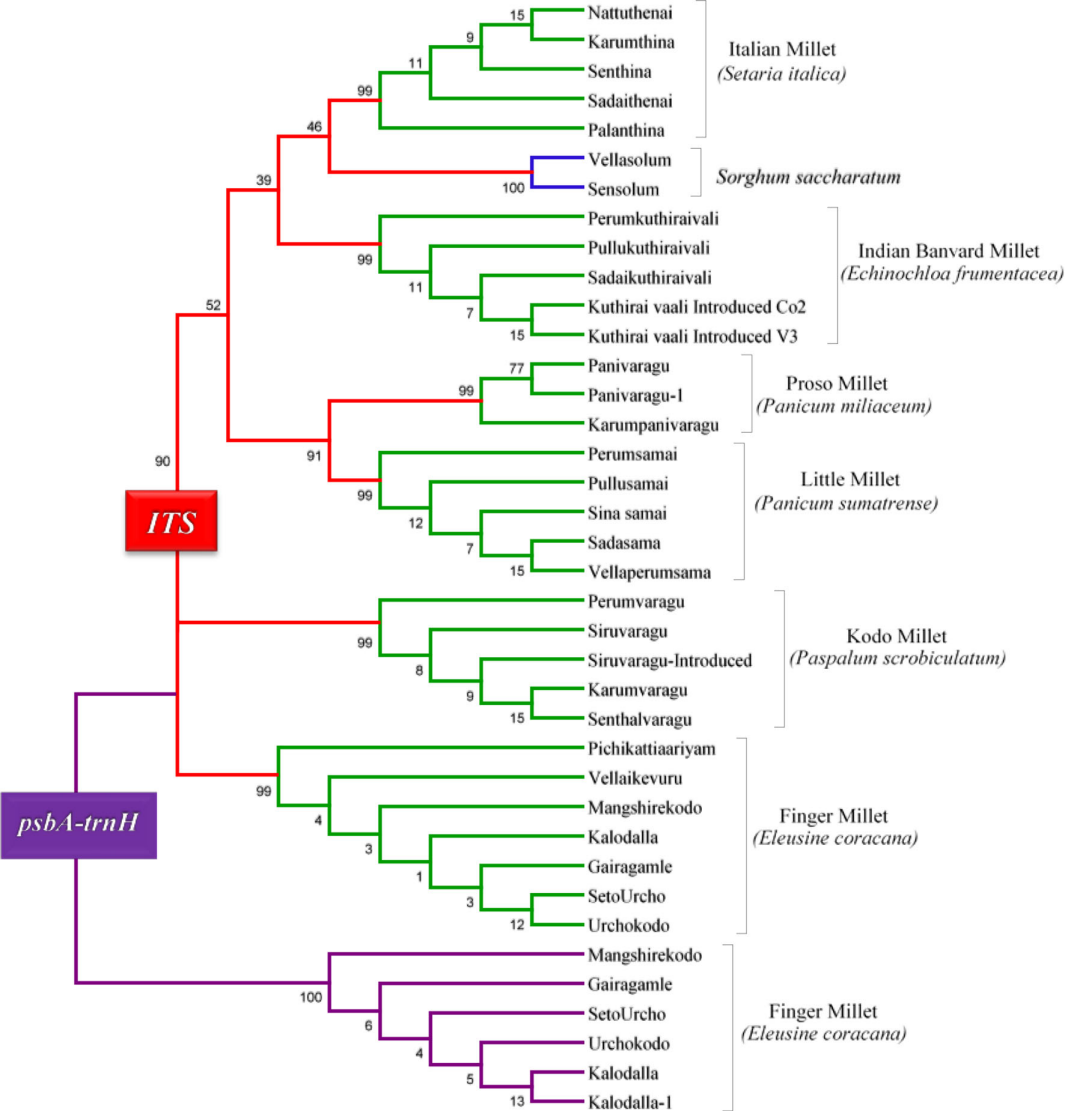
Neighbor-joining (NJ) tree based on the p-distance of the nuclear barcoding loci *ITS2* and *trnH*-*psbA*. Branch *color* represents scientific species (*red*) and landraces (*green*) based on farmers traditional knowledge (TK) of small millets. The branches in blue represent the major millet Sorghum. Neighbor-joining (NJ) tree of Finger millet (*Eleusine coracana*) landraces based on the p-distance of the chloroplast barcoding region *trnH*-*psbA*

This research advances knowledge of the variation among small millets. DNA barcoding of the nuclear region *ITS*-*2* region in our study revealed considerable genomic variation among 32 landraces that represent five species of small millets (*Echinocloa frumentacea, Eleusine coracana, Panicum sumatrense, Paspalum scrobiculatum, Setaria italica*) and one major millet *Sorghum saccharum* (Fig. 1). All the 32 individual landraces fell into distinct clades with bootstrap support values corresponding to the six millet species. Furthermore, the plastid region *trnH*-*psbA* revealed further genetic variation among five landraces (Mangshirekodo, Gairagamle, Setourcho, Urchokodo, Seltsodalla, Kalodalla) in *Eluecine coracana* (Fig. 1). Using a combination of *trnh*-*psbA* and *ITS2*, this study revealed that there is at least one nucleotide polymorphism between any two landraces.

Our research provides a molecular diagnostic tool for fast and accurate identification of millet landraces. We utilized a tiered approach using *ITS2* DNA barcode to make 100 % accurate landrace (32 landraces) and species (six species) assignments for all 160 blind samples in our study. Previous research (Newmaster et al. 2013a, b, c) has shown that *ITS2* was the most efficacious of all the barcode suggested barcode regions based on cost, lab resources, and ease of use. The cost to identify one sample was $15 CDN and the time to generate a barcode was 24 h, but this cost and time could have been lowered if we employed high-throughput genomic methods such as next generation sequencing. We suggest that DNA barcoding using a tiered approach with *rbcL* + *ITS2* will provide a measure of diversity in addition to delivering a commodity identification tool to ensure authentication and traceability of landraces, all while assembling a DNA-based model for conservation of genetic diversity and the associated biocultural diversity (TK) of millet.

### Trait variation among landraces of small millet

Landraces are associated with highly valued traditional knowledge concerning traits. These morphological traits such as grain size and plant height are important to crop yield. Other traits are associated with ecological tolerance with respect to the types of areas on the landscape where these landraces will grow and flourish. Farmers have selected for these traits over many generations and the variation among these traits reflects the different needs of farmers whom seek to grow a particular landraces of millet with respect to different types of sites. Many agricultural traits, such as length of the growing season, salt tolerance, and germination requirements, are highly variable among the different landraces. We visited farms where they grew millet at low and very high elevations (1000–1500 m) in very harsh conditions. Perhaps one of our biggest discoveries is the existence of a drought tolerant landrace, which is of considerable importance given recent changes in the local climate. The farmers told us that many landraces are vulnerable due to the recent adverse climate conditions such as recurrent drought (Kothari et al. 2005; Khoury et al. 2010). A changing climate has resulted in low yield of millet or even crop failure as the rain ends early in the season before flowering can occur. For these areas, it is desirable to conserve local germplasm that can mature within the range of the rainy season. Over the years, a large number of early/late-maturing millet landraces with various desirable traits have to be introduced and evaluated. This research is part of a long-term goal for conservation and molecular characterization of millet landraces and the associated traditional knowledge used in Indian agriculture. There is also considerable variation in millet landrace traits associated with cultural utility. This category of traditional knowledge includes traits such as nutrition, taste, amount and type of flour produced from grain, medicinal value, social status; some millet landraces are used to produce cakes for royalty.

**Fig. 2:**
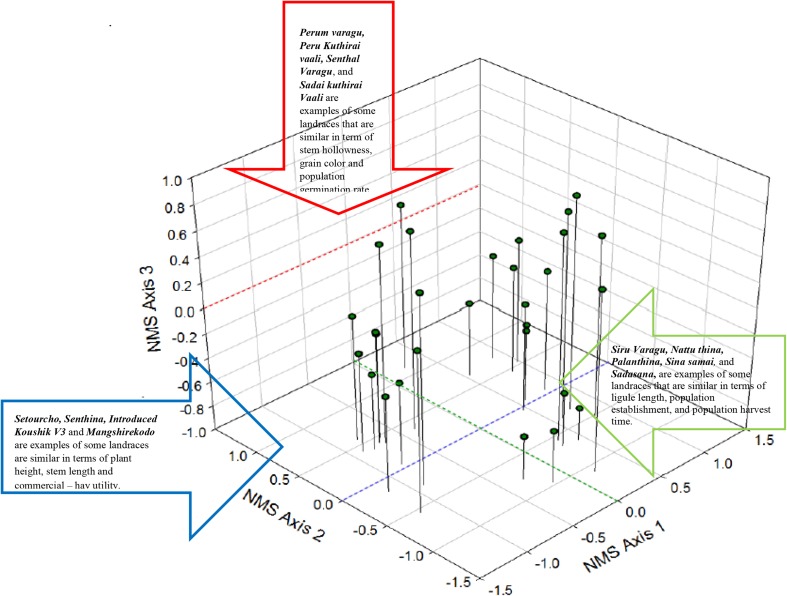
Non-Metric Multi-Dimensional Scaling was used to identify variation among 32 landraces and 50 agricultural traits (stress = 0.09)

In our study, there was considerable variation among the small millet landraces with respect to farmers’ traditional knowledge of agricultural traits. Non-metric multidimensional scaling was used to identify variation among 32 landraces and 50 agricultural traits (Table 2; Fig. 2). There was extensive variation in agricultural traits across NMS axis −1 (2.0 SD), NMS axis −2 (1.75 SD) and NMS axis −3 (1.22 SD). In total, 32 agricultural traits had significant correlations with NMS axis −1. Some examples are collar color, ligule length, ligule color and length, water needs, population establishment and harvest time (*r* > 0.7, *p* < 0.05). Most landraces that have yellow or brown colored collars are spread out on the right side of the axis and landraces with collar which are dull green or pale spread out on the left side of the axis (Fig. 2). Most landraces that have shorter, yellow and brown colored ligules on the right side of the axis and those that have longer brown colored ligules are spread out on the left side of the axis (Fig. 2). Almost all landraces that are spread out on the right side of the axis 1 have lower water needs than those found on the left side of the axis 1 (Fig. 2). Most landraces associated with long population establishment and harvest times are spread out on the right side of the axis 1 and most landraces that have low population establishment and harvest time are found on the left side of axis 1 (Fig. 2). Compared to NMS axis 1, there were fewer agricultural traits that had strong significant correlations with the NMS axis 2. In total, 26 agricultural traits had a significant correlation with NMS axis 2. Plant height, Plant biomass, Stem length, Straw yield and Straw hay utility commercial are examples of some traits that had strong significant correlations with NMS axis 2 (*r* > 0.6, *p* < 0.05). Most landraces that are on the upper area of axis 2 are shorter and have lower biomass than those on the lower area of the axis (Fig. 2). In addition, landraces on the upper area of axis 2 have shorter stem length and lower straw yields compared to the landraces spread out on the lower half of the axis (Fig. 2). Finally, landraces on the upper area of axis 2 have lower commercial hay utility potential than the landraces found on the lower portion of the axis (Fig. 2). Only 9 agricultural traits had a significant correlation with NMS axis 3. Grain length and population germination are examples of two traits that had strong significant correlations with NMS axis 3 (*r* > 0.5, *p* < 0.05). Landraces on the top portion of axis 3 had more round grains than those in the bottom portion of the axis. Most landraces on the top of axis 3 have lower population germination rate than the landraces spread out on the bottom portion of the axis (Fig. 2).

**Table 2:** Pearson correlation and significance (*p* < 0.05) with each of the Nonmetric Multidimensional Scaling (NMS) axis for 50 agricultural traits among 32 landraces based on native farmer’s traditional knowledge

Agricultural trait	NMS axis 1		NMS axis 2		NMS axis 3	
	Pearson correlation	Significance (*p* < 0.05)	Pearson correlation	Significance (*p* < 0.05)	Pearson correlation	Significance (*p* < 0.05)
Plant duration	−0.233	0.223	0.194	0.313	*0.401*	*0.031*
Plant habit	0.218	0.256	*0.456*	*0.013*	0.065	0.737
Plant height	0.205	0.286	*0.698*	*0*	0.257	0.178
Plant physique	*0.589*	*0.001*	0.294	0.121	0.19	0.324
Plant biomass	0.344	0.068	*0.773*	*0*	−0.107	0.582
Stem diameter	0.216	0.261	*0.601*	*0.001*	0.277	0.145
Stem length	0.24	0.209	*0.681*	*0*	0.383	0.041
Stem color	*0.572*	*0.001*	*−0.456*	*0.013*	*0.361*	*0.055*
Stem juice	0.236	0.218	−0.141	0.466	0.188	0.328
Stem hollow or heavy	0.252	0.187	0.234	0.221	−0.334	0.076
Stem Internode diameter	*0.439*	*0.017*	0.311	0.1	0.219	0.253
Stem Internode length	*0.385*	*0.039*	*0.568*	*0.001*	0.256	0.18
Stem Internode color	*0.556*	*0.002*	*−0.512*	*0.004*	−0.031	0.874
Stem Internode juice	−0.172	0.373	−0.103	0.594	0.205	0.287
Stem Internode hollow or heavy	0.008	0.966	*−0.532*	*0.003*	*0.428*	*0.021*
Stem sheath length	*0.662*	*0.001*	0.046	0.813	0.192	0.318
Stem sheath color	0.672	0	−0.462	0.012	0.343	0.069
Stem sheath blade	−0.632	0	−0.486	0.007	0.008	0.966
Collar length	0.493	0.007	0.329	0.081	−0.215	0.264
Collar color	0.787	0	−0.482	0.008	0.164	0.394
Ligule length	0.771	0	0.348	0.064	0.344	0.068
Ligule color	0.784	0	−0.45	0.014	0.147	0.445
Leaves position	−0.012	0.949	−0.118	0.541	−0.075	0.7
Leaf sheaths margins	−0.866	0	0.403	0.03	0.18	0.349
Leaf sheaths length	0.402	0.031	0.589	0.001	0.277	0.145
Grain color	−0.385	0.039	−0.159	0.409	0.52	0.004
Grain length	0.497	0.006	0.159	0.411	−0.702	0
Grain size	−0.032	0.87	0.642	0	0.256	0.181
Grain apex	−0.709	0	−0.109	0.572	0.088	0.651
Seed storage endurance	−0.555	0.002	0.273	0.152	−0.015	0.938
Seed storage without treatment	−0.529	0.003	0.375	0.045	−0.104	0.59
Seed storage with treatment	−0.251	0.189	0.439	0.017	−0.241	0.209
Seed preparation for storing	0.789	0	−0.246	0.198	−0.454	0.013
Seed preparation for sowing	0.799	0	−0.064	0.74	−0.172	0.371
Productive tillers	0.603	0.001	0.567	0.001	0.018	0.926
Population germination rate	0.1	0.606	−0.446	0.015	0.642	0
Population establishment	0.752	0	0.218	0.255	0.458	0.013
Population harvest time	0.748	0	0.368	0.049	0.087	0.654
Flowering time	0.36	0.055	0.596	0.001	0.191	0.32
Panicle density	0.101	0.602	0.221	0.25	0.348	0.064
Panicle yield	0.185	0.335	0.22	0.252	0.152	0.43
Grain Yield	0.143	0.461	0.101	0.602	0.332	0.078
Straw Yield	0.158	0.414	0.755	0	−0.11	0.57
Grain yield by season	0.406	0.029	0.429	0.02	−0.089	0.646
Grazing	0.424	0.022	−0.283	0.137	0.49	0.007
Best season	−0.34	0.072	0.144	0.457	−0.15	0.437
Water needs	0.888	0	0.044	0.822	−0.005	0.98
Ploughing needs	0.432	0.019	0.415	0.025	0.352	0.061
Hay utility commercial	−0.313	0.098	0.715	0	−0.098	0.614
Disease resistant	−0.833	0	0.268	0.16	0.06	0.759

Strong Pearson correlation and *p* values are in italics

### Traditional uses of small millets

The farmers’ traditional knowledge of small millet landraces also included a wealth of information. This included information concerning the utility of specific landraces with respect to variability in nutrition and medicinal value. However, we also learned about the social value of these millet landraces, which is deeply entrenched in the history and culture underpinning the need for conservation of biocultural diversity. Their small-scale commercial developments are incredibly important to individual communities and collectively as one culture. Theses farmers place a considerable amount of value in sustainable development. Some of the TK shared with us is discussed in the following paragraphs and documented in Table 1.

**Fig. 3:**
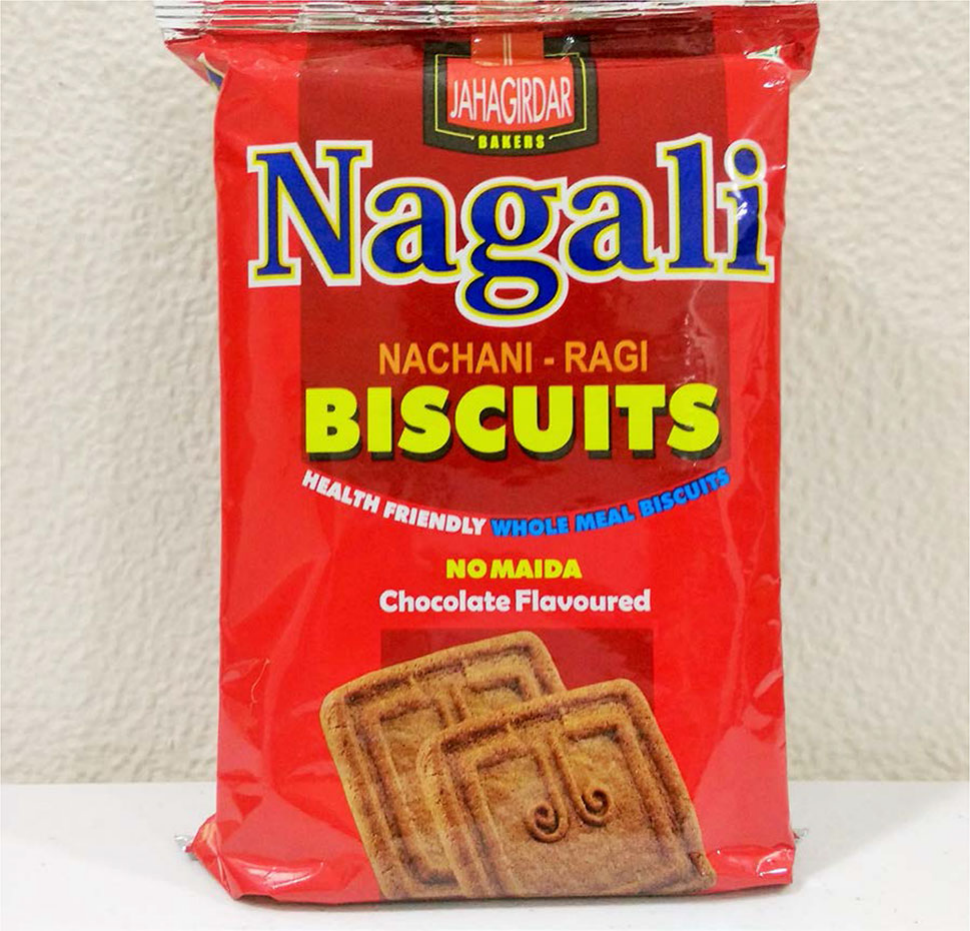
Industrialized millets cookies consumed by high end people for their brake fast

Local farmers emphasized the nutritional importance of all landraces of small millets. The traditional way of consuming millet products and the value of millet porridge called ‘Kapai’ as food had long been appreciated in their culture. Kapai (Ragi) is known to be high in protein, and a good source of starch, vitamins and minerals, such as iron, and phosphorus (Barbeau and Hilu 1993; Vadivoo et al. 1998). Fermented ragi is a good source of probiotics (Beghel et al. 1985; Antony et al. 1996). Ragi is regularly consumed by all ages and genders in our study with daily consumption by women and children due to its perceived nutritional value. Ragi porridge is breakfast staple for many households as the farmers claim that they feel very satisfied and strong after consuming the porridge from breakfast rather than other types of food. The starch found in millet is known to extend the duration of time for digestion, supplying a slow constant supply of energy (Dreher et al. 1984; Dharmaraj and Malleshi 2011). An elderly male farmer of Kaskikot Village Development Committee (VDC) of Dhading district in Nepal shared “When we eat food prepared from finger millet, we don’t feel hungry for a long time. We regularly serve our field workers *dhindo* (millet pudding) and *roti* (millet chapatti) in our community”. Similarly, another old female farmer in Dhikur Pokhari VDC of the same district revealed that consumption of finger millet-based food helps control obesity. In fact, many farmers claim to use millet to control diabetes due to its low glycemic load (Dreher et al. 1984; Dharmaraj and Malleshi 2011; http://www.fao.org/docrep/t0818e/T0818E0c.htm); these farmer are aware that the slow digestibility of millet sugar made it the perfect food for diabetic patient. Pregnant and lactating women in many households preferred a millet-based diet because it provided energy as well as prevented weight gain. They believed that during pregnancy, the consumption of millet helped induce lactation and maintain optimal body temperature and energy levels after delivery. Farmers told us that animals that consume millet-based feed have higher yields of milk; some of the feeds include fermented millet. We were told that human consumption of fermented ragi helps individuals to recover from stomach disorders caused by over consumption of liquor. This is very common as there are roadside shops that sell this product on most street corners in the morning (Patel et al. 2014) (Fig. 3).

Traditional knowledge revealed that millet landraces have medicinal value in all the communities we visited. In fact, there are many ailments that are treated using various landraces of small millet. For example, a common cold is treated with a mixture of hot ragi porridge with lemon juice and salt; variations of this recipe included the addition of garlic, ginger paste, *Timur* (*Zanthoxylum alatum*) paste or turmeric. Vitamin balls are commonly made from ragi mixed with other herbs to treat sick children and malnutrition. These balls are made from freshly matured seeds of *Keshvaragu*, which are crushed into a green paste with the leaves of *Modakathan* and other ethnotaxa such as *Thirivarugu*. This mixture ground up into a fine powder using a hand stone mill and then formed into small (about 1 cm in size) spherical balls and stored them in a cool place. Small millets can also be used as a poultice to treat insect bites, fevers, chickenpox, smallpox and measles. Farmers have actually made plaster cast for broken bones of the fractured human or animal body parts using millet flour plaster in the early days; some millet landraces make better cast that last longer such as the landrace *Paundure kodo*, which can be used to heal a fracture in 30–45 days. Millet landraces are also used veterinary applications to treat livestock. Most of the treatments are related to digestive disorders such as bloating and diarrhea. Even though, finger millet is stigmatized against its color and taste, as people feel ashamed to offer *dhindo* to their special guests or relatives, it is well appreciated for its medicinal value, as it helps relieve from cold, asthma, allergies, gastric, joint aches and diabetes (Dukpa 2012).

Millet landraces have considerable social value. The historical value of millet is well documented throughout India (Fuller 1999, 2002, 2003a, b, c, 2005a, b, 2006a, b; Weber and Fuller 2008). We provide here some local context shared by the farmers that have played a role in the social customs of their communities. In all the villages of Nepal we visited, there are many Hindu temples raised by the locals, of which the foundation for their main God idol-statue in the temple is formed to hold huge amounts of *kodo* grain (about a metric ton); the locals believe that their God idol will have more power. The *Gurung*, *Newar* and *Magar* communities considered small millet as a ‘pure’ grain with which to brew spirits that are offered to the Gods/Goddesses in various festivals or life ceremonies. Foxtail millet is one of the seven pure grains mentioned in Hindu manuscripts. In fact, they believe that evil can be chased away with the flames from this millet alcohol in a sacred process called *Chhyahore*. In Nepal, the *Chepang* community considers a landrace of barnyard millet to also be a ‘pure’ sacred grain of great religious importance, which is offered to their *Kuldevata* (God of the clan) in *Rishi Panchami* festival; food prepared from barnyard millet is consumed during a fasting period for well-being. Locally prepared finger millet alcohol is offered as *Sagun* to relatives and guests to honor them with respect. On contrary, finger millet is considered ‘impure’ grain and not suitable for religious ceremonies especially for *Brahmin* and *Chhetri* communities. We also learned of a common tradition where the majority of the farming community will donate millet seed and flour as a wedding gift—a gift of life. The amount of millet gifted depended on the wealth of the farmer. The value of millet in their community was celebrated in all ceremonies, as it is the life source of their culture.

**Fig. 4:**
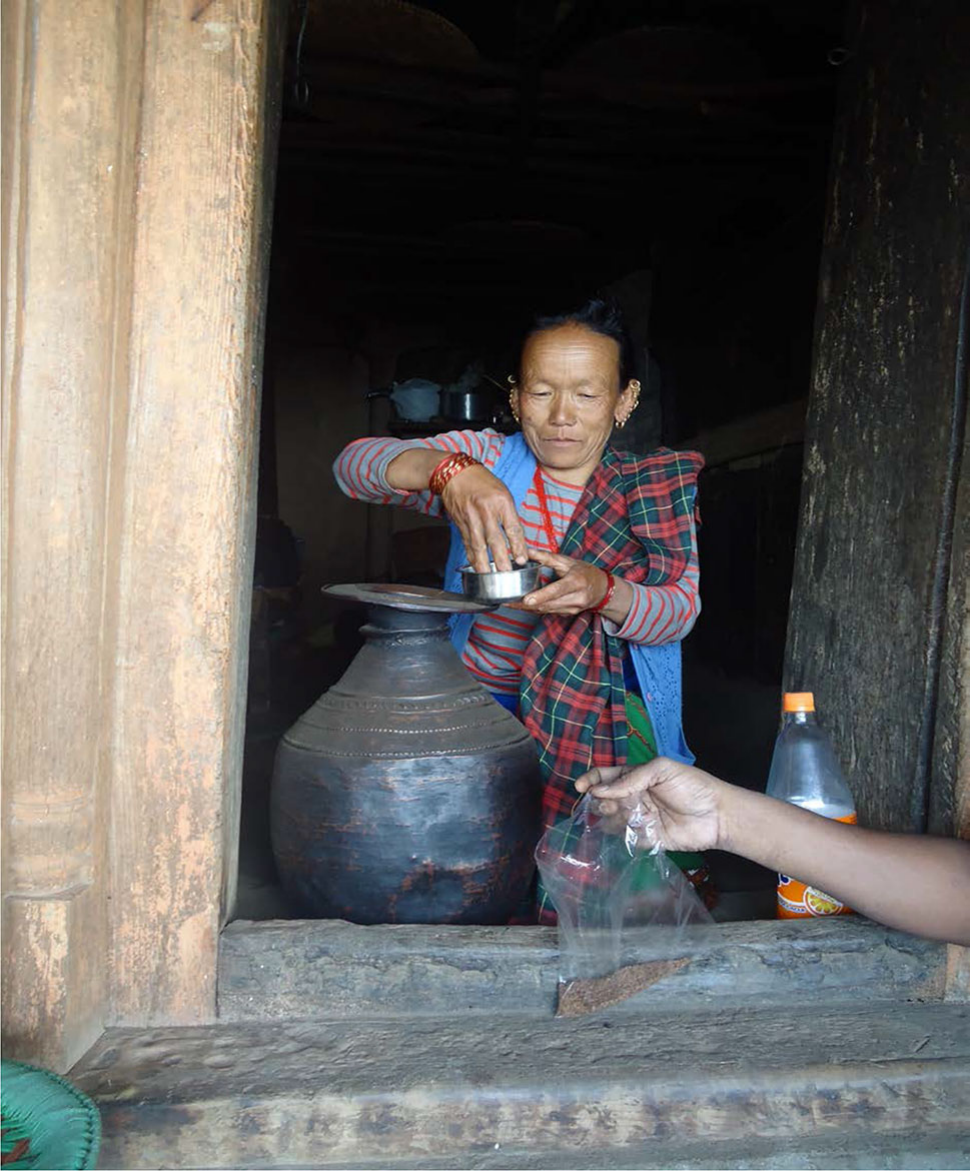
Farmers preferred Kalourcho ragi—Save this ragi for their own use (either bread making or local wine making)

Small millet is an important commodity in rural communities. The commercial value of the various landraces is considerable to several local industries. For example, different landraces of finger millet are among the major ingredients in cookie industry. The cookie industry (Fig. 4) provides annual revenue for local farmers through supply agreement contracts. In Nepal, finger millet is grown for local alcohol brewing industry to make special ragi malt-based wine that is popular within the community; each community has special recipes used for different occasions and celebrations. We learned of a local brewed wine called *Chhyang*, which was prepared during our visit to one of the study sites. The local process is rather innovative in that the local millet grains are boiled and fermented using special locally prepared yeasts. The fermented grains are stored in pots for one to three months and alcohol is prepared from of the fermented millet in an elaborate steam distillation apparatus. Farmers often prepare their own country liquor called *rakshi* using their own landrace of *kodo* with an inoculum, which is often uniquely chosen by the farmer (Fig. 4); we were shown one example of an inoculum prepared from a plant called *Buki phul* (*Anaphalis triplinervis*) using either its flower or root or stem. *Rakshi* is a mild tasting alcohol and the locals claim that it has medicinal and nutritional qualities, such as topical application to sooth aching joints or burns; relief from pain and fatigue; treating stomach disorders; and prevention of high altitude sickness. The stalks of millet are commonly used as thatching for roofs, and as straw for both feed and bedding of livestock. This straw is traded or sold within local communities and stored for long periods of drought.

Traditional farmers have many environmental uses for millet of which most apply to agronomy within their communities. All millets are used on steep slopes to control erosion and drought conditions. Several landraces in our study (e.g., *Thirikulasama* and *Pani varagu*) were used by farmers in dry steep slopes because they exhibit drought tolerance. The farmers told us that these are small millets that they seed by hand and they will germinate and grow without any irrigation; the farmers do not return to that field until they need to harvest. Although the yield from these drought tolerant landraces is low, the farmers are assured that there will be a harvest even in the dry slopes or in seasons with extend drought. Interestingly, we learned that Kodo millet hay is environment friendly as a biocontrol agent. Kodo millet straw is in high demand where the betel leaf (*Piper betel*) is grown. Betel leaf is a good cash crop, which is commonly affected by pathogenic fungi. Betel plant stem and root decaying disease causes major losses in yield for the betel plant growers. According to the betel farmers, mulching with kodo millet straw helps prevent the pathogenic fungi from establishing, resulting in much higher yields. We were also told that the straw from *Thirivarugu*, which is not palatable for animals, can be used as mulch around the beds of *Capsicum*, tomatoes, and eggplant. The mulch is prepared by making piles of this millet, and then by luring many millipedes and centipedes from the forest. The millipedes and centipedes converted the tough stems and leaves into a rich, humus-like mulch.

### Millet landrace conservation genetics and protection farmers’ rights

Local communities of farmers have been and should continue to be the stewards for the conservation of millet landrace genetics and the associated traditional knowledge. Although millet is currently undervalued as an agricultural crop, it has been historically recognized as one of the most important agricultural commodities as evidenced from numerous archaeological sites throughout the globe (Dida et al. 2008). There is a danger of losing the current TK of millet landraces if there is no immediate action to recognize local farmers’ knowledge in small millet crop breeding and on farm conservation programs. Their historical breeding programs have considerable influence on the selection of traits and the genetic diversity of landrace millets. Our surveys of marginal farmers who grow millet indicate that these rural men and women are the custodians of millet genetic resources and TK; they have retained seeds and TK for over 4000 landraces (Seetharam et al. 2006). Women play a key role in millet-based food production systems and insist that we must maintain a diversity of landraces to deal with complex environmental and health issues. They should have an active role in the conservation strategy for maintaining genetic diversity and TK of small millets. The Protection of Plant Varieties and Farmers’ Rights (PPVFR) Act provides a legal framework for local community farmer’s ownership of plant genetic resources in India (Swaminathan 2002). Consequently, the PPVFR Act has provided a registry mechanism local landraces cultivated by Indian farmers and communities and facilitates their claims for benefit sharing and recognition of their associated TK. The important next step in this process is to document genetic diversity using a common molecular methodology. We suggest that DNA barcoding will provide a measure of diversity while delivering a commodity identification tool to ensure authentication and traceability of landraces (Newmaster et al. 2013a, b, c). This provides a methodology for conservation of genetic diversity and the associated biocultural diversity (TK) of millet landraces still used in rural communities throughout Asia. As the demand for locally grown food from original or farmers’ landraces increases in domestic and international markets, small millets have excellent potential for commercialization. A unique DNA barcode could help in preventing the (illegal?) monopolization of landraces as an agricultural commodity by outside market forces, which contradicts local community practices and values and violates the PPVFR Act. Landrace DNA barcodes could be used to prevent fraudulent product substitution, which undermines the economy of local communities. The landrace barcodes could also help consumers in verifying authenticity of local product and its traceability through supply chain. The purity of landrace is an important attribute for consumers as well as farmers striving to maintain pure unadulterated landraces and the associated TK.

The need to develop a conservation strategy to protect genetic diversity and TK of landrace millets has gained a growing recognition in the international forums. Small millets are an important crop throughout Asia and Africa as recognized by the DFATD and UNESCO. To conserve this cultural heritage, there has been a recent effort to collect a large number of landrace accessions by the combined efforts of the All India Small Millets Coordinated Project (AISMCP) and International Crops Research Institute for the Semi-Arid Tropics (ICRISAT). The International Development Research Center (IDRC) has recently funded several projects in South Asia, under its Canadian International Food Security Research Fund (CIFSRF), to document indigenous landraces of small millets and associated indigenous knowledge system. Many of these accessions have been evaluated in the country and some were released as commercial cultivars for the highlands and lowlands. Still others have been used in supplementing the germplasm base of the international and national agricultural systems around the globe. Maloles et al. (2011) and Rengalakshmi (2005) illuminated the importance of the Indian Small Millets germplasm in the world collection as it relates to conservation of biocultural diversity as urged by the Convention of Biological Diversity and UNESCO’s ‘Man and Biosphere Programme’ and the Declaration on the Rights of Indigenous People. Farmers told us that in recent years, the diversity of landraces is decreasing due to reduced value within the Indian agricultural system (Kothari et al. 2005; FAO 2010; Khoury et al. 2010).

A strategy for the conservation of genetic diversity is not complete and several steps need to be completed. Although India has done excellent job in providing legal framework through PPVFR Act, it does not have a national DNA database on small millets that could be help in prioritizing scientific research and monitoring commercial use and conservation of landraces. Current research on Indian small millets relies on the allele frequency calculations on the western species and varieties using the NCBI GenBank. The ideas presented in this paper is the first step towards demonstrating the use of barcoding methodology and generating scientific data on small millets landraces cultivated in India and Nepal. Such research has to be scaled up by national research institutions to create complete database numerous landraces cultivated and practiced by local farmers. This database can help the government authorities to apply DNA as evidence in the adjudication of farmers’ rights cases through PPVFR. We suggest that our methods and preliminary research were used to establish a biological reference material (BRM) for important landraces of small millet in India and Nepal. The Wildlife Institute of India (WII) located in Dehradun has started the DNA-based Wildlife Forensics Services to provide scientific evidence on crimes that impact biodiversity (Gupta 1993; Gupta et al. 2005, 2006, 2011a, b). The WII forensic scientists for the purpose of identification of illegal trade could utilize the millet landrace DNA barcode BRM database. The development of a BRM library for small millets should be linked to TK and associated farmers rights as the next step in the conservation of this valuable food. In a time where food security issues are mounting, we feel that small millets are a valuable resource that needs to be conserved and sustainably managed so it may benefit society-at-large.

## Electronic supplementary material

Below is the link to the electronic supplementary material.

Supplementary material 1 (XLSX 56 kb)
